# The Role of Fungi in the Cocoa Production Chain and the Challenge of Climate Change

**DOI:** 10.3390/jof7030202

**Published:** 2021-03-10

**Authors:** Johannes Delgado-Ospina, Junior Bernardo Molina-Hernández, Clemencia Chaves-López, Gianfranco Romanazzi, Antonello Paparella

**Affiliations:** 1 Faculty of Bioscience and Technology for Food, Agriculture and Environment, University of Teramo, via R. Balzarini 1, 64100 Teramo, Italy; juniorbernardo.molinahernandez@studenti.unite.it (J.B.M.-H.); apaparella@unite.it (A.P.); 2 Grupo de Investigación Biotecnología, Facultad de Ingeniería, Universidad de San Buenaventura Cali, Carrera 122 # 6-65, Cali 76001, Colombia; 3 Department of Agricultural, Food and Environmental Sciences, Marche Polytechnic University, via Brecce Bianche, 60131 Ancona, Italy; g.romanazzi@univpm.it

**Keywords:** *Theobroma cacao*, fermentation, fungal diseases, cocoa, filamentous fungus

## Abstract

Background: The role of fungi in cocoa crops is mainly associated with plant diseases and contamination of harvest with unwanted metabolites such as mycotoxins that can reach the final consumer. However, in recent years there has been interest in discovering other existing interactions in the environment that may be beneficial, such as antagonism, commensalism, and the production of specific enzymes, among others. Scope and approach: This review summarizes the different fungi species involved in cocoa production and the cocoa supply chain. In particular, it examines the presence of fungal species during cultivation, harvest, fermentation, drying, and storage, emphasizing the factors that possibly influence their prevalence in the different stages of production and the health risks associated with the production of mycotoxins in the light of recent literature. Key findings and conclusion: Fungi associated with the cocoa production chain have many different roles. They have evolved in a varied range of ecosystems in close association with plants and various habitats, affecting nearly all the cocoa chain steps. Reports of the isolation of 60 genera of fungi were found, of which only 19 were involved in several stages. Although endophytic fungi can help control some diseases caused by pathogenic fungi, climate change, with increased rain and temperatures, together with intensified exchanges, can favour most of these fungal infections, and the presence of highly aggressive new fungal genotypes increasing the concern of mycotoxin production. For this reason, mitigation strategies need to be determined to prevent the spread of disease-causing fungi and preserve beneficial ones.

## 1. Introduction

*Theobroma cacao* L. is a tree native to the Upper Amazon basin that includes territories in Brazil, Ecuador, Peru, and Colombia. *T. cacao* (cocoa) was first cultivated in Mesoamerica by the Olmec and Maya civilizations and later by the Aztecs. Thanks to the pre-Colombian cultures, it spread throughout the world. The seeds of this fruit (cocoa beans) are used to make chocolate.

Cocoa cultivation depends on the farming method adopted. In general, seeds are planted in a seedbed and transferred to the ground. Although it is not very common, cocoa seeds are sown directly in the soil in some countries. Planting often occurs at the beginning of the rainy season so that the soil remains moist while the roots become firmly established. The cocoa tree needs shade for its protection, and normal development is favoured by high and constant relative humidity. To this purpose, sometimes banana plants, coconut trees, or other species are planted along with the cocoa. Depending on the variety, it will take three or four years until the cocoa tree produces its first fruits, and the maximum harvest is reached after six or seven years. Although the fruits mature throughout the year, in general, there are four harvests each year: a small harvest at the beginning and a large one at the end of each of the two rainy seasons. The cocoa tree produces fruits called pods containing pulp and raw beans, and each pod generally contains from 25 to 40 seeds or cocoa beans [1]. When harvested, the outer pod is removed from the tree to extract the beans. After removing the pod, the beans are subjected to a fermentation stage (an essential step in which mucilaginous pulp surrounding the seed is removed) and a drying stage before being sent for further processing. These are the most crucial production stages, where important cocoa changes occur, ensuring that cocoa gets its characteristic flavours and aroma [2,3].

Cocoa is cultivated in several tropical and subtropical zones around the world. The temperature ranges from 15 to 32 °C, usually at an altitude below 300 m.a.s.l., although in some ecosystems, it can reach 1100 m.a.s.l. For proper growth, the crop requires uniformly distributed rain throughout the year, ranging between 1500 and 3000 mm. The largest producers are the Ivory Coast, Ghana, Indonesia, Ecuador, Nigeria, Cameroon, and Brazil [4]. The International Cocoa Organization reported world productions of 4697 thousand tons during 2019/2020 and estimates that its production tends to increase [4]. It is a market that in 2019 moved more than 50,300 million dollars in raw cocoa and cocoa products [5].

Similar to other crops, cocoa plants and beans are exposed to contamination and colonization by different microorganisms during the sequential production steps (crop, harvest, fermentation, drying, and storage). Indeed, in all countries where cocoa is produced, weather and crop practices support fungal growth and, consequently, product quality deterioration. In general, cocoa production quality depends on the genetic type, natural conditions in the site where plantations and management are located, and the postharvest activities, including fermentation, drying, and storage. In particular, filamentous fungi may infect several stages in cocoa processing, and poor agricultural practice may have a marked influence on the quality attributes of cocoa [6].

This paper gives an overview of the different fungal species involved in cocoa production and their presence during preharvest and postharvest procedures and about the role of climate change in the disease distribution and cocoa production.

## 2. Preharvest

### 2.1. Field

As mentioned above, *T. cacao* crops are found in pantropical regions, which are characterized by having two periods of rain and two periods of drought in the year. Thus, environmental conditions, such as temperature and relative humidity, are the two key features affecting the final cocoa quality. In general, high relative humidity and temperatures favour fungal growth and contribute to the diffusion of cocoa diseases. The primary inoculum exists in plant parts and soil, where it overcomes the dry season, infecting plants when the environmental conditions are favourable. Some fungal pathogenic species are responsible for the *T. cacao* diseases from bloom to harvesting. Several diseases have a high potential to devastate global cocoa production, reducing it from ca. 20 to 80% if certain diseases are widely distributed. In fact, cocoa pathogens have a confined geographic distribution. In this context, Marelli et al. [7] recently highlighted that the long lag-time between breeding, planting and economic pod production, could further aggravate the spread and the management of some diseases. On the other hand, climate change can also modify plant diseases’ expansion and change the host’s resistance [8,9].

#### 2.1.1. Fungi Associated with Diseases of the Aerial Plant Parts 

**Figure 1:**
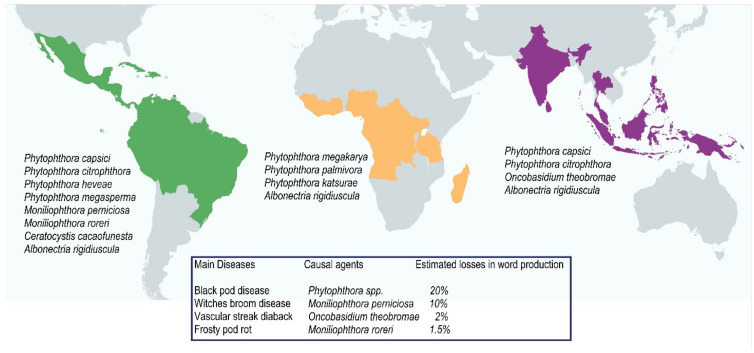
Main diseases in cocoa crops caused by fungi in the three cocoa-producing regions: America, Africa, and Asia and Oceania, related to the percentage of estimated losses in world cocoa production. Estimated values in secondary data [4,12].

Depending on the region where cocoa is grown, at least one or more of five diseases can cause severe losses. The agents of the diseases with the greatest impact are *Phytophthora* spp. (black pod disease), *Moniliophthora perniciosa* (witches’ broom), *Moniliophthora roreri* (frosty pod rot), *Ceratosystis cacaofunesta* (Ceratocystis wilt of cocoa or “Mal de machete”), and *Oncobasidium theobromae* (vascular streak dieback) [10,11]. In general, these pathogens are resistant to adverse environmental conditions. Figure 1 displays the main diseases in cocoa crops caused by fungi, which lead to the most significant economic losses to cocoa farmers in the three cocoa-producing regions.

***Phytophthora* spp.***Phytophthora* spp. belong to Oomycetes group and the Pythiaceae family. Some species of this genus cause the disease known as black pod rot (also known as *Phytophthora* pod rot, PPR), which is one of the most significant diseases that affect cocoa production. This disease was reported for the first time in Trinidad in 1727. *Phytophthora* spp. are also responsible for the leaf and nursery blights and stem canker.

Black pod rot economically represents between 20 and 25% of global losses of cocoa production [13]. In countries like Cameroon, the losses can reach the total production [14]; in some Côte d’Ivoire and Ghana farms, the mean annual pod losses are near 40% [15] if no control measures are taken. The organism can attack seedlings and different parts of the tree, such as flowers, buds, leaves, branches, trunk, and roots, but the pod’s suffer the main damage. In fact, *Phytophthora* spp. colonizes the internal pod tissues, alters the mucilage’s coloration and discolours, and withers the beans, causing the loss of the quality of the grains [13]. A brown spot is formed that eventually covers the entire pod, causing death. The survival of *Phytophthora* spp. in the soil and decomposing organic matter in the absence of their host is largely dependent on the two kinds of spores designed for long-term survival: chlamydospores and oospores. Furthermore, *Phytophthora* spp. produce swimming zoospores considered the principal dispersive agent of the infection [16]. In the species *Phytophthora megakarya* and *Phytophthora palmivora,* it is possible that genetic variations may occur, which help to overcome the resistance of the host, due to the formation of oospores starting from the two mating types of zoospores A1 and A2, when they are present.

The two main *Phytophthora* species that cause the disease are *Phytophthora megakarya* and *Phytophthora palmivora*. The first species is the most prevalent and aggressive and is considered the primary threat to cocoa crops in West and Central Africa [17]. *P. palmivora* is distributed worldwide, and in Cameroon and Ghana causes significant damage [16]. *P. megakarya* is reported as more virulent than *P. palmivora*, due in part to the fact that it is capable of releasing twice as many zoospores and because it does so more quickly after infection [18]. However, Ali et al. [19], suggested that *P. megakarya* is more aggressive probably because it produces appressoria more often than *P. palmivora,* allowing direct penetration while *P. palmivora* infects through stomata. 

Despite this, in a study conducted in six cocoa-growing regions in West Africa, where sixteen *Phytophthora* isolates were analysed, it was found that these isolates were more virulent and they reproduced faster than *P. megakarya* [20]. Recently, Morales-Cruz et al. [21] demonstrated that *P. megakarya* genome is extensive (222 Mbp) and nearly twice the size of *P. palmivora* (135 Mbp) and most known *Phytophthora* species (∼100 Mbp on average). Ali et al. [16] found that *P. megakarya* shows a different virulence-related gene complement, similar in size and potentially of greater diversity than *P. palmivora*. While specific genes can determine each species’ pathogenicity, the pathogenic potential of *P. palmivora* seems to have increased via whole-genome duplication or tetraploidy. On the contrary, broad gene duplication, especially among virulence-related gene families, possibly mediated by transposable elements, has expanded the pathogenic potential of *P. megakarya* [16].

Other species like *P. capsici* and *P. citrophthora* appear to be the dominant and most important species infecting cocoa in some countries of Central and South America, Indonesia, and India [22]. *Phytophthora heveae* also has been established to cause black pod disease in Mexico [13]. In addition, *P. katsurae* have been reported in Cote d’Ivoire [23] and *P. megasperma* in Venezuela [24]. Still, no economic losses due to their infection have been reported. Currently, the causative factors for the geographical distribution of the *Phytophthora* species are not yet understood [13], and more research is required on this topic.

The best conditions for the development and proliferation of black pod disease are rainfall, high moisture, and low temperature. In particular, the disease symptoms emerge two weeks after the onset of rains [25]. Although the factors contributing to support the maximum temperature growth in *Phytophthora* species have not been investigated in detail, it has been reported that *P. palmivora* is more resistant to high temperature than *P. megakarya* [20,26]. In addition to climatic factors, the disease’s manifestation and progression are determined by the cocoa genotype and the *Phytophthora* species involved [27]. For example, Amelonado-type Upper Amazon and Lower Amazon selections are apparently less susceptible to *Phytophthora* spp. than Criollo and Trinitario types, so they are used in breeding programs [28]. However, it is crucial to consider that an increase in infection rate could be a consequence of climate events such as floods.

*Moniliophthora perniciosa.* This basidiomycete is the causal agent of Witches’ Broom disease in cocoa. It is the main limiting factor for cocoa production in Brazil [29]. *M. perniciosa* (previously *Crinipellis perniciosa*) is a hemibiotrophic pathogen that presents two morphologically distinct life phases. In the biotrophic phase, the mycelium is monokaryotic and inhabits the intercellular space without anchoring and invades flower cushions, developing fruit and meristematic tissues. In this way, the plant develops irregular growth structures, including clustered shoots with atrophied internodes that look like a broom. In the necrotrophic phase, a dikaryotization process occurs, presenting two nuclei connected by a basidiomycete clamp in the cell. The mycelia invade and destroy the infected tissues, then the basidiocarps emerge from dead tissues and discharge spores that start a new infection [30,31]. The pathogenicity factor in *M. perniciosa* involves an enzymatically inactive chitinase (MpChi) that is highly expressed during the biotrophic interaction with the cocoa plant. Mutations suppress this chitinase’s enzymatic activity, but the enzyme retains its substrate-binding specificity and prevents chitin-triggered immunity by sequestering immunogenic chitin fragments [32].

The infection development depends on atmospheric humidity (rain, fog, dew, and relative humidity), notably water films on sensitive tissues or poorly drained soils maintaining a high-water content in the plants. Peaks in diseased fruits occur 5–6 months after peak flowering, which coincides with precipitation periods [33]. The formation and maturation of basidiocarps are stimulated at 20–25 °C and during the light day period, whereas they do not usually form above 30 °C. Likewise, basidiospores’ discharge is optimal at the same temperature and 80% relative humidity [34]. The basidiospores can germinate rapidly in water, about 30 min at 30–33 °C. The presence or absence of any environmental conditions affects the host phenology, the production of basidiocarps, the release of basidiospores, the dispersion, infection, and the synchrony between these events [35]. 

*Moniliophthora roreri.* Known also as *Monilia*, *Moniliasis*, *Nieve*, *Mancha Helada*, *Pasmo*, *Paludismo*, or *Pringue* [36]. This basidiomycete is the causal agent of frosty pod rot disease, restricted to the northern part of South America (Peru, Ecuador, Colombia, and Venezuela) and all of Central America [37]. It is possibly the biggest threat to cocoa production, particularly for Brazil and the producing countries of West Africa and Asia if it were to be presented. Although global losses are low, it is only because it is not present in the major bulk producing countries. The disease is so invasive that in a few years the outbreak in Central America reduced yields by near 90% [38].

*M. roreri* is classified as a hemibiotrophic pathogen, just like *M. perniciosa*. During the biotrophic phase, it is possible that the pod develops malformations and progresses quickly to the necrotrophic phase, where rot occurs. Finally, it produces sporulation on the surface [39]. *M. roreri* and *M. perniciosa,* are closely related. Their genomes are similar, including many of the genes considered to be important in the disease process. The *M. roreri* genome (52.3 Mbp) is larger than the *M. perniciosa* genome (44.6 Mbp) [40]. Relevant studies using molecular markers indicate that *M. roreri* propagates clonally [41] and expresses the hydrolytic enzymes chitosanases, lipases, and cutinases during the early stages of the interaction with *T. cocoa* pods [42].

Climatic changes (e.g., temperature regimes, atmospheric chemistry, and drought) may influence plant-pathogen interaction, altering the genetic resistance to cocoa moniliasis [33]. In this context, the disease progresses more rapidly in the warmest locations and is probably limited to intermediate humidity areas, with the wind being the principal natural dispersing agent. However, when the humidity is very high, the spores’ weight increases and they do not disperse easily [43]. Moreover, considerable day/night temperature differences favour the germination of pathogen spores. *M. roreri* has an extensive incubation and latency period (40 to 60 days). Warm temperatures (20 to 27 °C) and high relative humidity (80 to 100%) are appropriate environments for the germination of its spores and the fast fungal penetration [44]. Leandro-Muñoz et al. [44], applying statistical and mathematical modelling to determine the relationship between environmental conditions and *Moniliophthora* pod rot development, found that the fungal microclimatic requirements vary throughout the cycle, probably because *M. roreri* has a long latent period.

*Ceratocystis cacaofunesta.* It causes the “Ceratocystis wilt of cocoa” or “Mal de machete”, a severe Latin American disease. It is a member of the Latin American clade containing *Ceratocystis fimbriata* species, differentiated in the function of minor morphological differences, pathogenicity to cocoa, ITS-rDNA sequences, and intersterility [45].

The first reports of the disease occurred in Ecuador (1918), Colombia (1940), Trinidad (1950s), Venezuela (1958), and Costa Rica (1958). In the 1970s, it began to extend from Central America to Brazil (the southwestern Amazon (Rondônia) (1978) and in Bahia (1990s)) [46], where it is believed to have been introduced on plants that were witches broom resistant [47]. Genetic studies proved that the populations of *C. cacaofunesta* of Ecuador and Rondônia are native, and those of Colombia, Costa Rica, and Bahia are introduced populations [47].

*C. cacaofunesta* penetrates the cocoa tree through wounds caused by some insects or through infected tools used in the harvest (machete). It enters through the xylem where the chlamydospores germinate, invading the vessels of the xylem. Being a necrotrophic fungus causes the death of cells during colonization, where they obstruct water and nutrient transport, the plants turn yellow, then brown, wither, and die. It is homothallic and can reproduce sexually and asexually through vegetative propagation and conidial formation [48]. Occasionally, pods can be affected because the fungus can colonize their central vascular system without visible external symptoms. *C. cacaofunesta* has an optimum growth when the temperature is between 18 °C and 28 °C. In these conditions, the fungus produces ascospores in a week and is adapted to survive in the form of mycelium within the host or the form of aleurioconidia in different adverse environments such as soil or plant waste.

The biological and molecular mechanisms of this disease are scarcely documented in the literature. Recently, Lopez Molano et al. [49] provided evidence that phosphatidylinositol-specific phospholipases-C (PI-PLCs) could be very important for the pathogenicity of this species.

*Ceratobasidium theobromae.* Previously named *Oncobasidium theobromae* (syn. =*Thanatephorus theobromae*), it is a basidiomycete (Ceratobasidiales) that causes the disease known as “vascular streak dieback” (VSD) disease [50]. It is mainly found in Indonesia, Malaysia, and Papua New Guinea, from where it has spread to all cocoa-producing countries in South Asia. The fungus has a low sporulation rate. The basidiospores are produced after a long period of wetness and dispersed by the wind so that the symptom’s development is correlated with a rainy season during the previous months [51]. Formation and discharge of basidiospores occur mainly after midnight until the early morning when they lose viability due to morning sunshine exposure. Basidiospores are very sensitive and remain viable in the basidiocarp only for a few hours. When the basidiospores germinate, the hyphae penetrate soft tissues like unhardened leaves and branch termini through the cuticle. Guest and Keane [51] suggested that there is a relationship between infection periods and rainfall peaks, since long periods of wetness are required for the production, dissemination, and infection on the leaves. In this context, a high disease spread rate is reported in areas with rainfall exceeding 2500 mm a year [7].

It is possible that the levels of resistance found in several Upper Amazon and Trinitario genotypes reduce the future threat of the disease, mainly in Southeast Asia [52]. In Sulawesi, tests for resistance are also being carried out in high-yielding local cocoa clones and suggest strategies for incorporating VSD resistance [53,54]. Recently, Marelli et al. [7] highlighted that this pathogen could not move from the vascular tissue to the placenta and colonize the beans. For this reason, this fungus might not influence the quality of the cocoa products. Studies by Ali et al. [55] revealed that the *C. theobromae* genome presents a typical pathogenesis model, where the fungus secretes effector proteins involved in suppressing plant defence mechanisms as well as enzymes required for degradation of cell walls and other cell components.

*Albonectria rigidiuscula. Albonectria* (synonyms: *Calonectria* and *Nectria*) *rigidiuscula* (anamorph: *Fusarium decemcellulare*) causes two types of symptoms. One is cushion gall, which is a term that groups several different forms of flower cushion hypertrophy, five types of which have been identified and described (green point gall, flowery gall, knob gall, disk gall, and fan gall) in *T. cocoa* and other tropical trees [52]. The second type of symptom is dieback, where stag heads of debilitated branches develop [11]. It has been demonstrated that this disease is more common in stressed trees and in tissues that are damaged by insects.

A. *rigidiuscula* is important in Colombia (South America), in some countries of Central America such as Nicaragua and Costa Rica [56], and recently in Cuba [57], Africa (Nigeria, Ghana), and Sri Lanka [11]. The fungus is disseminated during the humid months of the year when the incidence is higher and the disease is also more evident [57]. This facultative parasite fungus is sometimes confused with other diseases, since it colonizes the plant right after an attack of *Phytophthora* spp., *Lasiodiplodia theobromae*, other plant pathogens, and/or insect damage, such as mealybug (*Pseudococcus njalensis*), and possibly others [56,58]. In cushion gall, the fungus is transported and introduced into the inflorescences and young beans, which develop galls that in some places are so numerous that they sterilize the tree. In dieback, the fungus develops different lesions unchecked in the debilitated tissue and causes chronic dieback [59]. *A. rigidiuscula* incidence is higher during the humid periods of the year.

Pink disease. This disease affects several tree species, including *T. cacao*. *Erythricium salmonicolor* (Syn. *Phanerochaete salmonicolor*, and *Corticium salmonicolor*) is recognized as its causative agent, although it is not very important worldwide. It has occurred in Ghana [60], Malaysia [61], and Western Samoa [62]. The Pink disease occurs mainly in the branches where there is an abundant growth of mycelium of salmon or pink colour, given by their fruiting bodies. After the branch’s death, the infection advances towards the tree’s bark, causing death if it is not controlled in time.

Other diseases. Some diseases such as anthracnose caused by *Colletotrichum gloeosporioides* or *Colletotrichum theobromicolum*, and Diplodia caused by *Diplodia theobromae*, *Lasiodiplodia theobromae*, among many others, have been little studied [63]. Although they can cause some diseases, their incidence is low due to the crops’ current management, the new existing varieties, and the applied control strategies. However, many of these fungi are part of the mycobiota and are reported as endophytes in cocoa.

#### 2.1.2. Fungi Associated with Root Diseases

The fungal soil populations interact with the roots of plants in the rhizosphere. These interactions can be detrimental or favourable. Some soil fungi are severe root pathogens or pathogens for the entire plant, and they can persist in the soil due to the presence of resistant spores. The main source of the fungal inoculum in cocoa comes from forest trees that are removed before starting to grow cocoa or when planting shade trees [52]. In general, all root diseases have similar symptoms, including rapid wilting of the leaves and the plant’s immediate death. These diseases can be identified by the appearance of fruiting bodies in the plant’s collar or the roots. In this review, we consider four widespread destructive root pathogens.

*Phellinus noxius* (*Fomes noxius*) is the cause of the disease known as brown root disease. This devastating disease affects various plant species, including *T. cocoa* [64]. *P. noxius* is a basidiomycete from the order Hymenochaetales, with optimal growth between 25 and 30 °C. It grows very well in very acidic soils, even at pH close to 3.5 [64] with a pantropic and subtropical distribution at elevations below 1000 m a.s.l. It is found mainly in Central America and the Caribbean, Africa, Asia, and Oceania [65]. The primary symptoms are yellowing of leaves, wilting, and defoliation; however, it is difficult to distinguish the disease’s early stages [66].

*Rigidoporus lignosus* (*Fomes lignosus*) causes white root disease. This rhizomorphic fungus has an ectotrophic growth habit. It develops in soils where rubber crops were previously grown; the infection occurs via wounds or after direct penetration of dead surface cells’ walls. The disease begins at the roots, destroying the root system until it reaches the collar region. At this point, when foliar symptoms appear, the death of the tree is imminent, and the fungus can remain in the soil and infect new plants [67].

*Rosellinia pepo* and *Rosellinia bunodes* are the agents that cause black root rot, also called Rosellinia root rot. It is distributed in tropical areas in Latin America and the Caribbean [68], but it is also found in West-Africa, the West Indies, and Asia. *Rosellinia* spp. are easily adaptable to low pH, which causes substantial cocoa mortality in acidic soils, especially those with low available phosphate and rich in organic matter [69]. Its presence is favoured in intermediate soil humidity levels. In Colombia, where *Rosellinia* root rot is severe, the disease is mostly associated with somewhat acid soils rich in organic matter with a high soil moisture level [69].

Moreover, it is a facultative parasite with more saprophytic than parasitic abilities. In general, they have smooth perithecia that produce unicellular ascospores [70,71]. The species *R. bunodes* and *R. pepo* are considered opportunistic pathogens, and some authors suggested that *Rosellinia* spp. act as a second invader after infection with *C. fimbriata* [71]. In a cocoa tree, the symptoms advance from the leaves to the root. The leaves initially become yellowish and dry, fall, then the branches dry, and finally, the tree dies [71].

*Armillaria mellea* (Physalacriaceae), is the agent of Armillaria root rot disease, or collar crack. This necrotrophic pathogen is found on soil with low pH and low nutrient availability in boreal, temperate, and tropical regions [72]. It has been reported mainly in several tropical Africa regions without presenting high economic impacts [73]. The majority of *Armillaria* species are bioluminescent, and form mycelial fans and rhizomorphs [74]. Although their fruiting bodies are edible, stomach discomfort can occur when they are not appropriately cooked [75]. Armillaria root disease is transmitted by reddish-black rhizomorphs, hyphae, and by contact with infected roots. The rhizomorphs adhere to the root, penetrate it, and the hyphae propagate through the phloem and the secondary xylem, colonizing the surrounding tissues and causing necrotic lesions. The symptoms include wilting, early senescence, leaf abscission, dieback, and rapid onset of death [76].

### 2.2. Epiphytic Fungi

Although not enough research has been done on the ecology of epiphytic mycoparasites in cocoa crops, Hoopen et al. [77] reported the presence of *Clonostachys* spp. as the most commonly isolated native mycoparasite, especially *C. byssicola*, *C. rosea*, followed by *Fusarium* spp. in cocoa trees. They also reported *Clonostachys* spp. for the biocontrol of black pod disease caused by *P. palmivora* and moniliasis caused by *M. roreri*.

### 2.3. Endophytic Fungi

The endophytic fungi from plants are non-pathogenic fungi that can promote plant protection, growth, and development by secreting some beneficial substances. They are taxonomically and biologically diverse from the host, but they can colonize internal plant tissues without producing lacerations. It is widely recognized that some endophytes can actively fortify their host plant against pathogens altering their response to diseases by either secreting bioactive compounds that eradicate or inhibit the growth or by inducing systemic resistance via the activation of the host plants’ endogenous signalling pathways.

The endophytes associated with cocoa show only some degree of host affinity. They are highly diverse and can be acquired from the environment [78,79]. The influence of endophyte infection is related to several factors, including canopy cover and leaf chemistry, among others [80]. It has been suggested that the different ways in which endophytic fungi develop their role in the plant include competition, antibiosis, and mycoparasitism. Hanada et al. [81] suggested that the co-evolution of cocoa and pathogens interactions, in the centre of origin of *T. cacao* (Upper Amazon region of Brazil, Bolivia, Peru, and Ecuador), has led to the co-evolution of associated endophytes that promote benefits to this crop. The same authors stated that *T. cacao* trees harbour diverse fungal endophytes due to being a tropical and perennial species.

The distribution of the endophytic fungi in the different plant organs varies both in abundance and species diversity. In general, a great part of the endophytic fungi in *T. cacao* belong to the Ascomycetes or their anamorphs, and only a limited number have been identified as Basidiomycetes. In this context, Crozier et al. [82], studying cocoa plants from natural forest and agricultural ecosystems in West Africa and Latin America, isolated fungal endophyte morphospecies from the stems and pods of *T. cacao*. Many of these isolates, analysed by sequence analysis of nuclear ribosomal DNA (rDNA), belonged to the Basidiomycota phylum, particularly to corticoid and polyporoid taxa. 

Although the composition and abundance of the species within endophytic communities are different depending on factors such as tissue, conditions around the plant [83], in *T. cacao,* it is possible to find at least two distinctive sets of endophytic fungi, one set in leaves [79] and another in stem [82,84] and pods [85]. The endophytes tend to be fungi such as *Colletotrichum*, *Botryosphaeria*, *Phomopsis,* and *Xylaria* that inhabit the leaves and branches. In contrast, the stem’s dominant endophytes tend to be in genera such as *Clonostachys* and *Trichoderma*, which generally are known as soil fungi [78]. In a very interesting study using DNA metabarcoding of the cacao tree leaves growing in five major cacao-growing regions in the central region of Cameroon, Wemheuer et al. [86] found that fungal endophyte community composition in the leaves, are affected predominantly by agroforestry practices and, to a lesser extent, by environmental factors.

As specified above, one of the roles of fungal endophytes in plant hosts is the ecological dynamics with pathogenic fungi. Field tests conducted by Hanada et al. [81] showed that strains belonging to the genera *Trichoderma*, *Pestalotiopsis*, *Curvularia*, *Tolypocladium*, and *Fusarium*, isolated from the stems and branches of cocoa, reduced the fraction of pods with symptoms of black pod disease caused by *P. palmivora.* On the other hand, *Colletotrichum gloeosporioides*, *Clonostachys rosea*, *Botryosphaeria ribis*, *Fusarium solani*, *Fusarium decemcellulare*, *Acremonium* sp., and *Xylaria* sp. found in leaves and pods, showed in vitro antagonism against *M. roreri* (frosty pod rot), *P. palmivora* (black pod rot), and *M. perniciosa* (witches broom) [78,87].

In addition, *Trichoderma* species isolated from roots and leaves of cocoa trees have shown prominent antifungal activities against some cocoa pathogens and subsequent disease through antibiosis, antagonism, mycoparasitism, and induced resistance. For example, *Trichoderma asperellum* introduced at the incision site in the bark for side grafting was efficacious in suppressing vascular streak dieback (VSD), as reported by Rosmana et al. [88]. In vitro studies have demonstrated that *T. asperellum, T. longibrachiatum,* and *T. virens* strains completely colonized and eliminated *P. tropicalis* and *P. palmivora* mycelium in precolonized plate assays [89]. Additionally, *T. asperellum*, *T. longibrachiatum*, *T. virens*, *T. harzianum*, *T. stromaticum*, and *T. asperelloides* have been isolated from cocoa trees and tested against *Phytophthora* spp. [89], *T. asperellum* against *Ceratobasidium theobromae* (VSD) [88,89]. Recently, the use of *T. asperellum* has been effective in the control VSD disease [90].

The potential of *Lasiodiplodia theobromae*, a foliar endophytic fungus, to control the growth of *M. roreri* and *M. perniciosa* was reported by [91]. Tondje et al. [92] documented significant advances in the search for endophytic fungi to be used as biocontrol agents of different diseases in Cameroon. They found that a strain of *T. asperellum* PR11 can be used to control *P. megakarya*. Another study was aimed to obtain stable formulations for biocontrol [93], while new species of *T. ovalisporum* with biocontrol potential were found in the Amazon basin of South America, a territory with high potential to drive new discoveries [94]. In addition, Villamizar-Gallardo et al. [95] suggested the potential use of *Botryosphaeria quercum*, closely associated with Platanus crops, which is commonly used in Colombia to shade cocoa plantation, and therefore are horizontally transmitted, i.e., through the environment, on *P. palmivora* and *M. roreri*.

### 2.4. Mycorrhizal Fungi

Arbuscular mycorrhizal fungi (AMF) establish symbiotic associations with the roots, enhance plant growth and yields, and induce systemic resistance against pathogens or adverse conditions. They do this by using various mechanisms and metabolic routes, including increased mineral nutrition (mainly phosphorous) and the expression of plant genes [96]. This is also observed with vesicular mycorrhizal fungi (AMF) [97] and some genus *Trichoderma* members.

In the cocoa crops (forest, agroforestry, and plantation), AMF diversity and community structure are strongly influenced by vegetation and ecological conditions, with lower diversity in natural ecosystems than the plantation [98]. In early studies, a mixture of AMF species of *Gigaspora* spp. and *Gigaspora margarita* spores gave the most vigorous growth and higher phosphorus content in the leaf tissues [97]. In addition, *Scutellospora calospora* and *Glomus mosseae* increased the phosphorus content of shoots [99]. More recently, [100,101], found that inoculation with the *Glomus* sp. and *Glomus mossae* promoted the cacao seedling growth in the greenhouse experiments.

On the other hand, *Acaulospora scrobiculata* was the main AMF associated with cocoa plants from northern Venezuela (Aragua, Miranda, and Sucre States) when the available phosphorus in soil was low [102]. The potential of AMF *G. margarita* and *Acaulospora tuberculata,* as well as the strain (PR11) of *T. asperellum*, promoted cocoa growth and induced resistance against *P. megakarya* in Cameroon [96]. Recent studies have shown the beneficial effects that AMF from Colombian cocoa soils to alleviate the stress that *T. cacao* plants show when translocating heavy metals such as cadmium [103]. In addition, *G. margarita* and *A. tuberculata* significantly reduced susceptibility to *P. megakarya* in the hybrid genotypes of the F79SA hybrid family of T. *cacao* [104].

## 3. Fermentation

Fermentation is an essential step for the development of the flavor precursors and for the final acidity of the cocoa beans. During this phase, microbiological and biochemical changes occur. Among the biochemical changes, the appearance of brown pigmentation due to phenolic compounds is an indicator of the fermentation of the cocoa bean, together with the content of the sensory precursors such as polyphenols, alkaloids, and volatile acidity. In general, after opening the cocoa pods at the plantation, the collected fresh cocoa pulp-bean mass undergoes a spontaneous fermentation for several days (2 to 7 d), depending on different parameters such as the variety of the cocoa plant, the climate, the local practices of fermentation (including heaps, wooden fermentation boxes, canoes, barrels, or baskets), the frequency of bean mixing or turning, the volume of cocoa to ferment, and the maturity and the sanitary conditions of the beans [3].

It is well known that healthy cocoa pods contain beans and pulp that are microbiologically sterile. Thus, all the microorganisms that drive the fermentation process derive from the environment. During fermentation, the pectinaceous pulp surrounding the beans is digested by microbial activity. Such activity generates metabolites and conditions that kill the cocoa embryos and trigger changes in the physical and chemical environment within the pulp-bean mass, important for the beans’ flavor and color. Yeasts, lactic acid bacteria, acetic bacteria, *Bacillus* spp. and molds carry out their activity in a well-defined temporal succession during fermentation.

In particular, yeasts eliminate the pulp that surrounds the fresh cocoa beans and depolymerizes or breaks pectin. Moreover, in the anaerobic conditions that prevail in the environment, yeasts carry out the fermentation of sugars to produce ethanol. On the other hand, bacteria ferment sugars and produce lactic acid, acetic acid, and mannitol. A correct fermentation, which is essential to obtain the full flavor of chocolate, cannot occur without the participation of these microorganisms.

Concerning filamentous fungal growth, it is important to consider that the conditions inside the bean mass, such as high amounts of alcohol together with low pH, organic acids, elevated temperatures, and microaerophilic conditions, are restrictive for these types of microorganisms. However, some studies reported their presence on the surface, especially in the last days of fermentation [105,106] or when the cocoa mass is not turned regularly [107]. The populations of the fungi during fermentation can vary according to the metabolites that develop within the fermentation mass and can be present in the order of 10^2^–10^3^ CFU/g at the beginning of fermentation, increasing to 10^6^–10^7^ CFU/g by 24–36 h, after which they are non-detectable [108]. Sometimes, fungi are present in deficient fermentation processes that give rise to sweet mucilage, which contributes to filamentous fungi development [109]. It is important to highlight that cocoa fermentation is carried out by populations specific to geographic regions (Table 1), which seem to have been adapted to a particular environment [110]. However, differences in the mould contamination level seem to be linked to the meteorological differences between cocoa growing and pod integrity, and to a lesser degree to a delay in pod opening, a factor that affects fungal diversity.

Although bacteria and yeasts’ role during cocoa fermentation has been clarified, the role of filamentous fungi in cocoa fermentation is not well understood. Since filamentous fungi can provide extracellular enzymes and help degrade the components of the mucilage, it could be possible that they play an important role in the development of other microorganisms, as well as on the cocoa quality. In this regard, Souza et al. [115], studying forty-six fungal strains isolated from soil and samples of cocoa, observed that all the strains were positive for pectinase activity and that 20% presented considerable pectinolytic activity. Lopez and Dimick [116] reported that *Aspergillus wentii*, *A. versicolor* and *Penicillium purpurogenum* isolated from cocoa beans presented polygalacturonase activity. All of the isolates had extracellular proteolytic and amylolytic reactions. In addition, Ogundero [114] isolated the filamentous fungi *Thermoascus aurantiacus* (thermophilic), *Mucor pusillus*, and *A. fumigatus* (thermotolerant), indicating that these fungi can develop in the fermentation medium above 40 °C and present considerable lipolytic activity, causing changes in the free fatty acid content in the fermentation medium. However, whether these fungal enzymes are produced and are active under the conditions of fermentation has not been studied yet. Another study revealed the fungi’ potential in cocoa fermentation to produce polygalacturonases, proteases, and amylases in East Java, Indonesia [108].

Several factors mark the growth of undesirable fungi during fermentation. One first factor is the low temperatures in the anaerobic stage (early two days), which can occur when the bean content in the fermentation box is low, makes the heat generated by the fermentation dissipate rapidly. In this case, the temperature does not rise sufficiently to inhibit fungi’ development, since at least 50 kg of biomass are required to reach an optimum temperature of 40 °C at this stage. A second factor is the lack of anaerobiosis due to air entry into the fermentation box or dehydrated beans when the pods are harvested long before shelling. Thirdly, in the aerobic stage (third day onwards), the fungal growth is marked by the permanence of cocoa beans in the upper level of the box for a long time where the relative humidity is lower, allowing for the development of fungi over other species of microorganisms [117].

One of the health risks associated with mould growth is the production of mycotoxins, particularly ochratoxin A (OTA) in cocoa beans, which has been associated with various types of cancer in rats, mice, and humans. The main fungal sources of OTA in cocoa beans are some species of the genus *Aspergillus* and *Penicillium*, but also black aspergilli (*A. carbonarius* and *A. niger* aggregate) in African countries and South America [118]. It is well known that defects and anomalies in cocoa pods strongly influence the physicochemical characteristics and occurrence of OTA in the final product. Contamination by spores that can generate OTA occurs mainly during the cocoa pod’s opening process that is done inside the crop without any protection.

The occurrence of OTA and the prevalence of OTA-producing fungi were reported in Bahía Brazil, together with the occurrence of aflatoxins and aflatoxigenic fungi (Copetti et al., 2011b, Copetti et al., 2014). A study conducted in Cameroon to assess how filamentous and toxigenic fungi were affected by the type of cocoa postharvest treatment (boxes or heaps) reported a large increase in filamentous fungi species at the end of fermentation [109]. Recently, Fonseca et al. [119] reported the contamination of aflatoxin and ochratoxin in 134 samples from 13 cocoa clones grown in the south of Bahia (Brazil): 38% (range between <LOD and 17.795 μg kg^−1^) were contaminated with aflatoxins, while 18% with OTA in the range of <LOD–274.90 μg kg^−1^. An adequate fermentation step in which the production of acids, mainly acetic acid which suppresses the growth of ochratoxigenic fungi, is very important for cocoa quality.

Another important health issue linked with cocoa fermentation is biogenic amines production, in particular histamine and tyramine. Even a small amount of these compounds can be harmful to susceptible consumers, but their presence is often unavoidable in fermented foods [120]. Although some authors reported the presence of tyramine, 2-phenylethylamine, tryptamine, serotonin, and dopamine [121,122], the contribution of the different fungal species have been not explored yet. Recently, we investigated the contribution of fungi to the production or degradation of biogenic amines and found that some fungi isolated from cocoa samples have amino-oxidase activity and other amino-decarboxylase activity [123]. These activities are closely related to biogenic amines present in cocoa.

## 4. Drying

The cocoa drying process can be carried out by solar drying or by artificial dryers and this is the final stage of the postharvest. In this stage, humidity is reduced to 7%, the volatile acidity content decreases, and the oxidation of polyphenol is stopped [124]. Overall, this production step promotes the biochemical and microbiological changes needed to enhance the quality of cocoa and prevent unwanted fungal growth.

The cocoa drying process can be carried out by solar drying or by artificial dryers, mainly hot wind systems depending on the farm’s technology. Artificial dryers reduce the drying time by keeping the relative humidity low and the temperature high and constant during the process. Some studies on kinetics and the transport phenomena of the drying of the grain’s internal and external water concluded that the best temperature for drying it is 30 to 40 °C. These temperatures coincide with solar drying’s thermal conditions, crucial for the development of different aromas and flavours in cocoa beans [125]. It is important to underline that there are no studies on artificially dried beans and fungi development during artificial drying. It is possible that the rapid change of a_w_ and the low relative humidity, as well as the higher content of volatile acids compared to sun-dried beans [126], do not allow their development.

In solar drying, the beans undergo different temperature and relative humidity changes, depending on the solar cycle and environmental conditions. The microbiota dynamics change slowly (seven days on average), providing favourable changes in the matrix, such as lower volatile acidity. This stage is very susceptible to the development of xerophilic fungi that prefer substrates with low water activity and can produce mycotoxins. Since sun drying is an outdoor process, cocoa can be contaminated with vectors such as insects, birds, and rodents that carry fungi that were not present in the fermentation stage. Thus, fungal contaminants in dry cocoa beans are commonly found. Therefore, prolonged drying increases the chance of fungal growth and spoilage. It has been reported that high contamination of cocoa beans by moulds may cause an increase in free fatty acids (FFA) beyond 1.75%, compromising the quality of cocoa butter [127]. In addition, in a significant percentage of the cases, mould contamination can challenge product safety due to mycotoxins’ presence.

Mould species that are usually present in sun-dried cocoa beans include different genera, with the predominance of *Aspergilli* (species *candidus*, *carbonarius*, *flavus*, *fumigatus*, *niger*, *nomius*, *melleus*, *parasiticus*, *tamarii*, and *westerdijkiae*) *Penicillia* (*species citrinum*, *crustosum*, *paneum*, and *sclerotiorum*), *Paecilomyces variotii*, and less frequently *Fusarium* spp., *Scopulariopsis* spp., *Geotrichum* spp., *Mucor* spp., *Rhizopus nigricans*, *Pseudopithomyces palmicola*, *Simplicillium* spp., *Talaromyces atroroseus* [109,112,127,128]. Different studies have demonstrated that during the later drying stages, there was an increase in potentially toxigenic species such as *A. flavus*, *A. parasiticus*, *A. niger*, and *A. carbonarius* [6,128].

Studies on sun-dried cocoa beans from Sierra Leona (Forastero variety), Equatorial Guinea (Amazon Forastero variety), and Ecuador (Amazon–Trinitario–Canelo Amazon hybrid) focused on identifying the sources of aflatoxins and cyclopiazonic acid (CPA) and OTA in these countries, found that 64.1% of 214 isolates of *A. flavus* produced aflatoxins and 34.2% CPA. Moreover, a high percentage of black *Aspergilli* (*A. niger* and *A. carbonarius*) strains (49.2% out of 138) were able to produce OTA [129]. A variety of fungi including the ochratoxin producers *A. carbonarius*, *A. niger* aggregate, *A. ochraceus*, *A. melleus*, and *A. westerdijkiae,* as well as the aflatoxins producers *A. flavus*, *A. nomius*, and *A. parasiticus* in addition to *Absidia corymbifera*, *Aspergillus* sp. nov. (related to *A. tamarii*), *P. paneum*, *A. candidus*, and *Eurotium chevalieri* were found in cocoa sun-dried beans collected at different times in Brazil [112,113,130].

Aflatoxin and ochratoxin are the most frequently occurring mycotoxins in fermented cocoa and dry beans. However, very recently, the nephrotoxic citrinin, which was not previously reported in cocoa beans worldwide, was detected in samples from Nigeria. The same authors indicated that the strains of *P. citrinum* isolated showed a high potential to produce citrinin in amounts up to 372 mg/kg [130].

From the literature, it is evident that the presence of mycotoxins can increase during the drying process due to inappropriate procedures, while materials (rack table, black tarpaulin, or concrete floor area) and platforms for the drying of fermented beans do not seem to favour mycotoxin production in cocoa beans [131].

## 5. Storage

**Figure 2:**
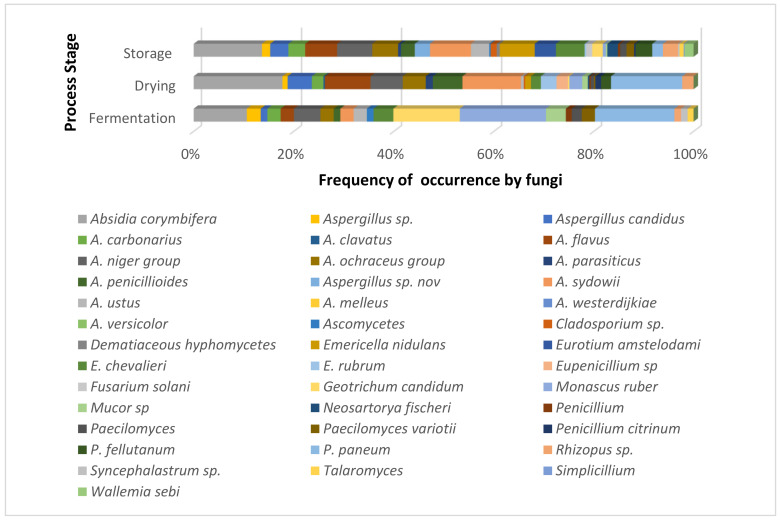
Main fungi found in the fermentation, drying, and storage stages during cocoa processing carried out by farmers on the farm [112,128,130].

During the cocoa beans storage and/or transport from the place of origin to the factory, it is likely that the grains harbour the fungi acquired in fermentation or drying (Figure 2) [112]. Poor postharvest management and inadequate drying, and poor storage conditions, can lead to rapid and effective invasion of stored cocoa beans by moulds. It is probable that fungi that can remain latent under ideal storage conditions can sometimes activate their metabolism if the grains’ humidity is higher than 8% due to poor drying conditions, water falling directly on the grains, or relative humidity greater than 80% during long periods of storage. This metabolic activity of some fungi can trigger the production and accumulation of mycotoxins in cocoa beans. In fact, mycotoxins will increase if inadequate storage conditions are employed, although they will tend to be reduced during roasting [106].

Although fermented and dry cocoa beans can be stored for up to 12 months under optimal conditions [132], the physicochemical changes in beans during transportation [133] or prolonged storage have not been investigated yet. Some sensory characteristics, such as colour and flavour, can change even under the best conditions, like beans moisture levels of approximately 7%, well-aerated and moisture-free environments [130], and a combination of the factors that favour a low water activity such as ambient relative humidity and temperature [134].

Airborne contamination is the primary source by which fungal spores can be transmitted from different media to the final product during storage. In a study conducted with commercial samples of cocoa from Sierra Leona, Equatorial Guinea, and Ecuador, the authors isolated mainly *Aspergillus* section *Flavi* (*A. flavus* and *A. tamarii*), *Aspergillus* section *Nigri* (*A. niger* aggregate and *A. carbonarius*), other *Aspergillus* (*A. fumigatus*, *A. nidulans*, *A. ochraceus*, *A. terreus*, and *A. versicolor*), *Penicillium* (*P. citrinum*, *P. commune*, *P. chrysogenum*, *P. glabrum*, *P. griseoroseum*, *P. olsonii*, *Eupenicillium cinnamopurpus*, and *Eupenicillium tropicum*), and others (*Chaetomium globosum*, *Cladosporium oxysporum*, *Emericella rugolosa*, *Eurotium amstelodami*, *Eurotium chevalieri*, *Nectria haematococca*, *Mucor racemosus*, *Phoma glomerata*, *Phoma medicaginis*, and *Rhizopus oryzae*) [129]. The high diversity of fungi observed due to the substrate’s ideal conditions suggests the need to implement or improve storage conditions to avoid contamination.

Mycotoxigenic fungi’s presence was also reported in samples from Ivory Coast and Nigeria, stored in bags for 6 to 12 months. In particular, species such as *Rhizopus stolonifer*, *A. niger* aggregate, *A. flavus*, *P. citrinum*, and *A. carbonarius* were found, being only *A. niger* able to synthesize OTA [135].

In a study carried out in Ivory Coast [136], the relationship between the quality of cocoa beans and the level of fungi contamination was sought, using the level of fermentation contrasted with the number of fungi that can cause the decrease in the quality. They found six species: *Absidia corymbifera*, *Rhizopus oryzae*, *Aspergillus tubingensis*, *A. tamarii*, *A. flavus*, and *Penicillium chrysogenum*. Furthermore, Copetti et al. [137] reported that the spectrum of the fungi isolated from samples stored up to one year in Brazil, was equal to those found in fermentation and drying. In particular, they isolated xerophilic *Eurotium* species such as *E. amstelodami E. chevalieri* and *E. rubrum*, *A. penicillioides*, *Cladosporium* sp., *Emericella nidulans*, *Eupenicillium* sp., *P. fellutanum*, and *Wallemia sebi*.

While the detection of aflatoxin and ochratoxin A in stored samples is common, on the other side, citrinin is not reported. This could be due to the cocoa microorganisms’ degradation, which may include diverse yeasts and bacteria with the ability to degrade citrinin [130,138].

## 6. Products

For the manufacturing of processed cocoa products, dry cocoa must be subjected to the roasting process. The beans’ temperature rises (120–145 °C) until its humidity is reduced to close to 2%. This increase in temperature allows the shell to be removed from the beans and drastically decreases the number of fungi. Therefore, the amount of fungi that passes to the products is very low, and in some cases, it can be influenced by the factory’s hygienic practices during processing. In addition, Fahrurrozi et al. [139] found that seed cotyledons contain proteins that may be involved in fungal protection. Therefore, the number of fungi that manage to colonize beans decreases.

Few studies evaluated the number of fungi in commercial products, although mycotoxins’ possible presence in these products is of fundamental importance [140]. In a study conducted in Nigeria, fungi of the genera *Aspergillus*, *Penicillium*, *Mucor*, and *Rhizopus* were found in samples of cocoa powder used to prepare a local drink [141].

The high level of mycotoxin contamination that could exist in chocolate is a severe health risk that should be urgently considered by the authorities. For consumer safety, necessary measures must be enforced in all processing steps to eliminate the level of mould contamination.

## 7. What about Climate Change?

**Figure 3:**
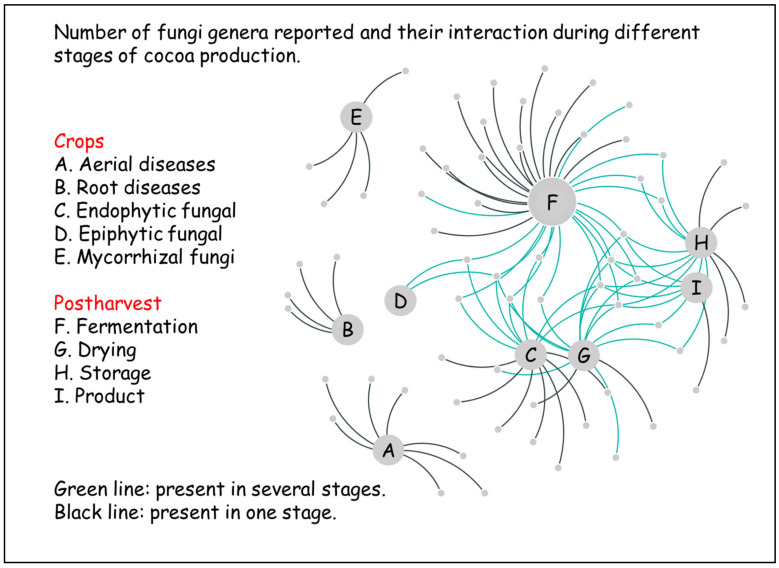
Main fungi interaction throughout the cocoa production chain.

It is generally known that the effects of climate change (CC) on agriculture include changes in levels of CO_2_, ozone, and UV-B that can modify plant diseases by changing host physiology and resistance [142]. In particular, CC includes changes in rainfall patterns, drought, flooding, and temperature that may influence disease epidemiology and/or modify the present land use for food crops, resulting in new pathogen disease complexes [143]. According to the Intergovernmental Panel on Climate Change (IPCC), in the coming years, CC will affect especially the tropics and subtropics, where precipitation will decrease at low altitudes and increase at higher altitudes [144]. Although CC’s impact will vary from region to region, according to the scenarios predicted for the regions where cocoa is produced, higher temperatures, more prolonged droughts, and increasingly frequent and strong storms are predicted to aggravate the current challenges faced by the agricultural production systems [144]. The extreme changes might shape crops and ultimately the yield of cocoa. In a very interesting review, Lahive et al. [145] considered the current research on cocoa’s physiological responses to CC. In the present paper, we consider the influence of CC on cocoa mycobiota during the different production steps because it is possible that new interactions of the fungi may affect the cocoa production chain and the final product’s quality (Figure 3).

Variation in temperature or altered precipitation may result in changes in cocoa pathogens that alter disease incidence and severity. Velásquez et al., [146] suggested that CC may (i) alter the stages and rates of development of pathogens and pests; (ii) accelerate the evolution of pathogens; (iii) reduce incubation periods; (iv) facilitate the introduction of invasive alien species, their establishment and diffusion; (v) change the physiology of the host–pathogen/pest interaction; (vi) produce changes in the geographical distribution of pathogens and pests; and (vii) affect production and consequently the socioeconomic variables. In this context, Bucker Moraes et al. [33] underlined that CC could induce significant risk on increases in moniliasis (produced by *M. roreri*), as literature shows the correlation between the germination of the disease’s fungal spores and precipitation, which is the only method for infecting other trees. In addition, the highly productive cocoa regions are profoundly affected by shifts in climatic regimes during the El Niño (ENSO)–La Niña (LN) cycle, which can favour fungal pathogenic infection in the productive and vegetative cycles of the cocoa trees [147]. Indeed, ENSO was responsible for the pod losses due to the increase of witches’ broom severity caused by *M. perniciosa* in the last five years. In this context, Gateau-Rey et al. [148] reported an increase in the pod losses from 2015 (15%) to 2017 (35%) during the drought.

CC has the potential to increase the incidence of pests and diseases, and introduce new types that find a favourable environment in the cocoa farm [149]. Researchers have considered that drought stress is beneficial to opportunistic fungal pathogens that may not otherwise impact crop hosts [150]. According to Kubiak et al. [151], climate change and global warming are not the only factors predisposing the roots of weakened trees to *Armillaria* infections, but also bacteria and fungi, as well as macro, meso, and micro-organisms growing in the soil around root systems, could enhance the proliferation of the pathogen and decrease the immune barriers in roots.

Although it is already accepted that CC modifies the distribution of phytopathogenic moulds, it is difficult to calculate all the effects also because there is a complete lack of information on host and pathogen adaptation to CC and accurate predictive models still do not exist for many diseases. As a result, evaluating of the possible impact of CC on the cocoa production chain should be treated with attention [142]. In particular, there are limited reports on the CC effects on cocoa fungal pathologies, although modelling studies have provided realistic scenarios on some plant diseases. For example, Ortega Andrade et al. [152], by using species distribution models (SDM) with nineteen climatic variables for the present and the future (5, 35, and 65 years), analysed the impact of CC on the potential distribution of *M. roreri* and *T. cacao* in South America. Their results suggested that the precipitation during the wettest month is the most influential variable for the presence and proliferation of *M. roreri*, and they estimated that this phytopathogenic fungus could extend from southern Ecuador to regions interconnected by cocoa crops in South America (Colombia, Venezuela, Peru, Bolivia, and Western Brazil). On the other hand, de Oliveira et al. [153] suggested that fungal communities in tropical grassland soils have greater sensitivity to drought than to temperature, which might increase the incidence of certain soilborne diseases.

The ability of fungal endophytes to confer stress tolerance to plants may provide a novel strategy for mitigating the impacts of global climate change on agricultural plant communities [154].

There are no studies on the impact that CC could have on cocoa mycorrhizal fungi. However, Bae et al. [155] showed that *T. hamatum* improved the tolerance to water scarcity of the cocoa seedlings colonized by this endophytic fungus. Recently, Bennett and Classen [156], examining the response of both mycorrhizal fungi and the associated plants, found that mycorrhizal fungi’ promotion of stress tolerance should allow temporal space for plant adaptation to CC. On the other hand, Kivlin et al. [157] suggested that leaf endophytes also respond to global change and improve the effects of drought in their host plants.

Although there is no information available on CC impact on cocoa fermentation, it is important to highlight that changes in temperature influence all the microbiota associated with fermentation (yeasts, bacteria, and filamentous fungi), which dominate in the cocoa seeds as they undergo continuous physical and chemical changes. Speaking about filamentous fungi, they are usually in low counts during fermentation due to the restricted conditions such as the ethanol and organic acid production and high temperatures that can rise above 45 °C after 48 h. As above mentioned, two scenarios could be present in the future: (I) increase of mean and maximum temperatures and drought, (II) periods of intense rainfall. Assuming scenario I, the fungi diversity could be decreased, with a selection of particular strains with particular technological properties that may not necessarily confer valuable cocoa characteristics. Paterson and Lima [150] proposed that existing thermotolerant and thermophilic fungal species will dominate and produce a variety of secondary metabolites and also mycotoxins. With scenario II, the fermentation might be extended, leading to a rise in bacteria of the genus *Bacillus* and in filamentous fungi that could cause off-flavours and the formation of mycotoxins, including the ochratoxigenic species *A. carbonarius* and *A. niger*.

Although some authors suggested that hot countries may produce safer food under CC because mycotoxigenic fungi will be inhibited [158], experimental data showed that the drying period is critical to avoid the formation of mycotoxins in the cocoa beans [131]. Indeed, some strains of *A. niger* can grow at 41 °C, showing a higher xerophilic ability compared to *A. carbonarius* and *A. ochraceus* [159]. Moreover, Moretti and Logrieco [160] suggested that CC may induce the presence of new fungal genotypes with high aggressiveness, increasing the concern of mycotoxin production.

## 8. Concluding Remarks

Fungi associated with the cocoa production chain have many different roles. They have evolved in a varied range of ecosystems in close association with plants and various types of habitats, affecting nearly all the cocoa chain steps (Table 2). The species causing diseases in cocoa crops are the primary source of economic losses to producers. Although the development of some fungal species is limited to specific regions, as for *M. perniciosa* in South America, it seems that the conditions under which fungi can develop would allow them to spread to other cocoa-growing regions if there is no adequate control in the propagation of seeds, cuttings, or plants that can carry fungal spores. Furthermore, CC could influence plants, making them more susceptible to fungal infection, favouring the spread of some fungal diseases, or changing the geographical distribution of phytopathogenic moulds. However, some risks linked to CC are more likely to be a problem in some regions than in others.

The study of fungi’s beneficial role in cocoa cultivation is focused on endophytic fungi that can help control some diseases caused by pathogenic fungi, particularly biocontrol agents made from endophytic fungi. In this regards some studies suggested that under changing climate scenario the use of fungal endophyte with commercial pesticide treatment could contribute to reduce the multiple disease resistance.

However, more studies on the role of fungi during fermentation are needed. It has been proved that they are present, but their specific role in the biochemical transformations of pulp and grains, the production of enzymes, and the interactions with other microorganisms at this stage still needs to be uncovered. Although researchers have agreed that a bad harvest leads to defects along all the postharvest process, there are no reports on how some fungal diseases could affect the cocoa quality.

On the other hand, it can be expected under strong selective pressure created by CC, that the distribution of heat-tolerant and heat-sensitive species change, creating conditions for the diffusion of more thermotolerant species able to produce mycotoxins, which can be found from the fermentation stage to the final product, with a significant risk for consumers’ safety. In fact, although a large part of the mycotoxin content is generally found in the roasted shell that is removed from the nibs, particularly ochratoxin A and aflatoxin can be detected in the processed products (roasted cocoa, nibs, butter, cocoa powder, and chocolate spread), and their can potentially increase with the CC.

## Figures and Tables

**Table 1:** Fungi reported in stage fermentation, classified by country.

Country (Region)	Fungi	Reference
Brazil (eastern Amazon region)	*Thielaviopsis, Fusarium, Aspergillus, Colletotrichum, Penicillium, Nigrospora, Hyphopichia, Trichosporon, Cophinforma, Cladosporium, Trichoderma, Agaricus, Talaromyces, Porobeltraniella, Neopestalotiopsis, Paecilomyces, Clonostachys, Lasiodiplodia, Purpureocillium, Cylindrocladiella, Wallemia, Nectria, Arthrinium, Curvularia,* and *Rhizomucor*	[111]
Brazil (Igrapiúna, Bahia)	*A. heteromorphus*	[2]
Brazil (Bahia)	*A. carbonarius* and *A. niger* aggregate	[112]
Brazil (Bahia)	*A. flavus* and *A. parasiticus*	[113]
Cameroon	*A. versicolor, Mucor* spp., *A. niger, Geotrichum* spp., *A. fumigatus, Fusarium* spp., *Rhizopus nigricans, A. tamarii, Syncephalastrum racemosum, P. sclerotiorum, A. flavus, Trichoderma* spp., *A. versicolor, Scopulariopsis* spp., and *P. crustosum*	[109]
Indonesia (East Java)	*P. citrinum, A. versicolor, A. wentii,* and *P. purpurogenum*	[108]
Nigeria	*Thermoascus aurantiacus* (thermophilic), *Mucor pusillus*, and *A. fumigatus* (thermotolerant)	[114]

Table 1 shows the fungi that have been found in different investigations during the fermentation stage. The presence of fungi in Brazil’s eastern Amazon region was determined by using High-throughput DNA sequencing (HTS).

**Table 2:** Summary of the genera reported of fungi involved in the different stages of cocoa production.

	Crops					Fermentation	Drying	Storage	Product
	Diseases of Aerial Plant Parts	Root Diseases	Endophytic Fungi	Epiphytic Fungi	Mycorrhizal Fungi				
Fungi reported in several stages			*Aspergillus* spp.			*Aspergillus* spp.	*Aspergillus* spp.	*Aspergillus* spp.	*Aspergillus* spp.
						*Cladosporium* spp.		*Cladosporium* spp.	
			*Clonostachys* spp.	*Clonostachys* spp.		*Clonostachys* spp.			
	*Colletotrichum* spp.		*Colletotrichum* spp.			*Colletotrichum* spp.			
			*Fusarium* spp.	*Fusarium* spp.		*Fusarium* spp.	*Fusarium* spp.		
			*Curvularia* spp.			*Curvularia* spp.			
						*Paecilomyces* spp.	*Paecilomyces variotii*		
			*Penicillium* spp.			*Penicillium* spp.	*Penicillium* spp.	*Penicillium* spp.	*Penicillium* spp.
			*Trichoderma* spp.			*Trichoderma* spp.	*Trichoderma* spp.		
	*Lasiodiplodia* spp.		*Lasiodiplodia* spp.			*Lasiodiplodia* spp.			
						*Geotrichum* spp.	*Geotrichum* spp.		
						*Mucor* spp.	*Mucor* spp.	*Mucor* spp.	*Mucor* spp.
						*Nectria* spp.		*Nectria* spp.	
						*Rhizopus* spp.	*Rhizopus* spp.	*Rhizopus* spp.	*Rhizopus* spp.
						*Scopulariopsis* spp.	*Scopulariopsis* spp.		
						*Talaromyces* spp.	*Talaromyces atroroseus*		
						*Wallemia* spp.		*Wallemia* spp.	
							*Absidia* spp.	*Absidia* spp.	
							*Eurotium* spp.	*Eurotium* spp.	
Fungi reported in a single stage	*Albonectria rigidiuscula*	*Armillaria mellea*	*Acremonium* sp.		*Acaulospora* spp.	*Agaricus* spp.	*Pseudopithomyces palmicola*	*Chaetomium globosum*	
	*Ceratocystis cacaofunesta*	*Phellinus noxius*	*Botryosphaeria* spp.		*Gigaspora* spp.	*Arthrinium* spp.	*Simplicillium* spp.	*Emericella* spp.	
	*Erythricium* spp.	*Rigidoporus lignosus*	*Chrysosporium* spp.		*Glomus mosseae*	*Cophinforma* spp.		*Eupenicillium* spp.	
	*Moniliophthora* spp.	*Rosellinia* spp.	*Pestalotiopsis* spp.		*Scutellospora calospora*	*Cylindrocladiella* spp.		*Phoma* spp.	
	*Oncobasidium theobromae*		*Phomopsis* sp.			*Hyphopichia* spp.			
	*Phytophthora* spp.		*Tolypocladium* spp.			*Neopestalotiopsis* spp.			
			*Xylaria* sp.			*Nigrospora* spp.			
						*Porobeltraniella* spp.			
						*Purpureocillium* spp.			
						*Rhizomucor* spp.			
						*Syncephalastrum racemosum*			
						*Thermoascus aurantiacus*			
						*Thielaviopsis* spp.			
						*Trichosporon* spp.			

## Data Availability

The data presented in this study are available on request from the corresponding author.
